# Gram-Negative Bloodstream Infection: Implications of Antimicrobial Resistance on Clinical Outcomes and Therapy

**DOI:** 10.3390/antibiotics9120922

**Published:** 2020-12-18

**Authors:** Majdi N. Al-Hasan

**Affiliations:** 1 Department of Medicine, Division of Infectious Diseases, University of South Carolina School of Medicine, Columbia, SC 29209, USA; majdi.alhasan@uscmed.sc.edu; Tel.: +1-803-540-1062; Fax: +1-803-540-1079; 2 Department of Medicine, Division of Infectious Diseases, Prisma Health-Midlands, Columbia, SC 29203, USA

The age- and sex-adjusted incidence rate of Gram-negative bloodstream infection (GN-BSI) is 84.5 per 100,000 person-years in the USA [1]. Recent advancements in diagnostics as well as clinical and antimicrobial management have reduced the overall case-fatality of GN-BSI to 12–15% [2,3]. GN-BSI accounts for 279,000 cases and 33,500–41,900 deaths annually in the USA based on the current population. Source control and early initiation of appropriate empirical antimicrobial therapy remain the most important modifiable variables for improving the clinical outcomes of patients with GN-BSI [4,5,6]. However, increasing antimicrobial resistance rates of Gram-negative bloodstream isolates continues to pose serious challenges to patients, clinicians, and researchers.

This Special Issue on Gram-negative bloodstream infections in *Antibiotics* highlights the impact of antimicrobial resistance to first-line agents on the clinical outcomes and antimicrobial management of GN-BSI. The authors of the studies in this Special Issue examine the effectiveness of different antimicrobial treatment strategies in BSI due to Gram-negative bacteria with various beta-lactam resistance mechanisms. They assess the implications of fluoroquinolone and beta-lactam resistance on the clinical outcomes of patients with GN-BSI. This is particularly important given that beta-lactams and fluoroquinolones represent the cornerstones of empirical and definitive antimicrobial therapy for GN-BSI, respectively [7,8,9]. The recently proposed definition of difficult-to-treat resistance (DTR) by Kadri and colleagues further enhances the importance of resistance to first-line agents (e.g., beta-lactams and fluoroquinolones) [2,3,7]. Review articles in the Special Issue discuss the role of novel antimicrobial agents in the treatment of BSI due to Gram-negative bacteria with DTR. The results of many investigations in this Special Issue have major implications for antimicrobial stewardship practices.

Previous studies reported that patients with fluoroquinolone-resistant GN-BSI had higher mortality and longer hospital length of stay than those with fluoroquinolone-susceptible bloodstream isolates [10,11]. Notably, most fluoroquinolone-resistant bloodstream isolates, particularly *Escherichia coli* ST131, carried antimicrobial resistance genes to other antimicrobial classes and were hence considered multi-drug resistant (MDR) [11,12]. Suzuki and colleagues went one step further and demonstrated that fluoroquinolone resistance even in the absence of concomitant phenotypic beta-lactam resistance still predicted higher mortality in BSI due to *E. coli* and *Klebsiella* species [13]. This remarkable work was the first to show the negative impacts of isolated fluoroquinolone resistance on patients’ clinical outcomes [13]. In this Special Issue of *Antibiotics*, Suzuki and colleagues demonstrate that patients with hospital-onset *E. coli* and *Klebsiella* species BSI due to either fluoroquinolone or extended-spectrum cephalosporin-resistant isolates have considerably longer hospital length of stay than those with BSI due to susceptible isolates [14]. The results of this multicenter matched cohort study argue that investments in antimicrobial stewardship and infection prevention are clearly justified based on the massive clinical and financial burden of fluoroquinolone and extended-spectrum cephalosporin resistance on the healthcare system in the USA [14].

To ensure maximum efficacy, the Clinical Laboratory Standards Institute (CLSI) followed the recommendations of the United States Committee on Antimicrobial Susceptibility Testing (USCAST) and lowered the minimum inhibitory concentration (MIC) susceptibility breakpoints of fluoroquinolones for Enterobacterales and nonfermenting Gram-negative bacilli [15]. This change has major implications on antimicrobial management, since fluoroquinolones remain by far the most commonly prescribed agents for definitive therapy of GN-BSI [8,9]. Shealy and colleagues precisely quantified the potential impacts of changing fluoroquinolone susceptibility breakpoints in Gram-negative bloodstream isolates [16]. An additional 5% and 8% of patients with BSI due to Enterobacterales and *Pseudomonas aeruginosa*, respectively, would not be eligible for fluoroquinolone definitive therapy after the implementation of the new CLSI susceptibility breakpoints [16]. The study calls for clinical pharmacokinetic and pharmacodynamic investigations to optimize the dosing of oral beta-lactams in this patient population and development of novel oral antimicrobial agents to fill this gap [16].

The MERINO trial provided much-needed clarity on the most appropriate antimicrobial therapy for BSI due to extended spectrum beta-lactamase (ESBL)-producing Enterobacterales [17]. However, antimicrobial management of BSI due to chromosomally mediated AmpC-producing Enterobacterales (CAE) remains controversial [18]. Previous literature demonstrated the effectiveness of cefepime in comparison to carbapenems for the treatment of BSI due to these bacteria [19]. In this Special Issue, Derrick and colleagues suggest that ceftriaxone may be a treatment option in patients with low-inoculum BSI due to *Enterobacter cloacae* and other CAE [20]. This is conceivable, since low-inoculum sources of GN-BSI are associated with lower mortality [21,22]. This multicenter cohort study is the first to report the effectiveness of ceftriaxone for BSI due to CAE [20]. The study has major antimicrobial stewardship implications, since it allows de-escalation of antimicrobial therapy from carbapenems or cefepime to ceftriaxone in patients with uncomplicated CAE BSI secondary to urinary tract source without obstruction or central venous catheter infection after catheter removal [20]. The results should not be extrapolated to patients with more complex sources of GN-BSI, such as pulmonary infections or multi-loculated intra-abdominal abscesses, due to the increased risk of resistance development and mortality [21,22,23,24].

The Special Issue includes another study that has huge antimicrobial stewardship indications. Lee and colleagues are the first to report the effectiveness of cefazolin definitive therapy for community-onset BSI due to susceptible Enterobacterales [25]. Data have been lacking on this topic due to the frequent updates to cefazolin MIC susceptibility breakpoints by various agencies. The results of this matched cohort study encourage antimicrobial stewardship practice of the de-escalation of antimicrobial therapy from broad-spectrum agents to intravenous cefazolin in patients with cefazolin-susceptible Enterobacterales BSI based on contemporary CLSI breakpoints [25]. This is crucial, since early de-escalation of antimicrobial therapy reduces the risk of *Clostridioides difficile* infection in these patients [26].

While original clinical research studies dominate this Special Issue, it has a fair share of high-quality basic science and translational research articles. Advancements of whole-genome sequencing (WGS) techniques have contributed to a better understanding of antimicrobial resistance mechanisms and improvement of antimicrobial therapy. Shelenkov and colleagues use WGS to define antimicrobial resistance mechanisms and virulence profiling of predominantly MDR *Klebsiella pneumoniae* isolates in the Russian Federation [27]. The incorporation and visualization of genotypic and phenotypic resistance patterns on one platform represents phenomenal work and sets a high standard for future investigations. Moreover, the authors describe two new multi-locus sequence typing (MLST)-based sequence types of *K. pneumoniae* [27]. In the second basic science article of this Special Issue, Fokam and colleagues examine iron chelation in murine models of systemic inflammation induced by Gram-positive and Gram-negative bacterial toxins [28]. This is a timely topic, given the recent development of cefiderocol, a novel antimicrobial agent for the treatment of Gram-negative bacteria with DTR that utilizes the iron transport system.

In addition to original research studies, the Special Issue includes two comprehensive reviews of the management of Gram-negative bacterial infections. Bassetti and colleagues summarize various beta-lactam resistance mechanisms and discuss antimicrobial treatment options for BSI due to Gram-negative bacteria with DTR [29]. The authors objectively review novel antimicrobial agents, examine the activity of these agent for various antimicrobial resistance mechanisms, and provide insight into the role of these agents in clinical practice [29]. Since the respiratory tract is one of the most common sources of Gram-negative bacteria with DTR, Martin-Loeches summarizes current concepts in the management of community-acquired and ventilator-associated pneumonia in intensive care units [30]. This is a timely review as well, given recent updates to international management guidelines on these two topics [31,32].

We hope this Special Issue of *Antibiotics* will enhance the knowledge and understanding of researchers and practitioners, improve clinical practice, and incite future innovative basic science, translational, and clinical research on antimicrobial resistance and GN-BSI.
